# Molecular Docking Analysis of Four Drugs (Phenytoin, Amoxicillin, Aceclofenac and Ciprofloxacin) and Their Association With Four Human Leukocyte Antigen (HLA) Alleles

**DOI:** 10.7759/cureus.62269

**Published:** 2024-06-12

**Authors:** Radhakrishnan Narayanaswamy, Divya Rajagopal, Vasantha-Srinivasan Prabhakaran

**Affiliations:** 1 Biochemistry, Saveetha Medical College and Hospital, Saveetha Institute of Medical and Technical Sciences, Chennai, IND; 2 Pharmacology, Saveetha Medical College and Hospital, Saveetha Institute of Medical and Technical Sciences, Chennai, IND; 3 Bio-informatics, Saveetha School of Engineering, Saveetha Institute of Medical and Technical Sciences, Chennai, IND

**Keywords:** toxicity (tox) analysis, cutaneous adverse drug reactions (cadrx), stitch, docking, good health and well-being, human leukocyte antigen (hla)

## Abstract

Background

Numerous reports have shown the role of human leukocyte antigen (HLA) alleles in the induction of cutaneous adverse drug reactions by moderating drug metabolism. We therefore aimed to investigate the docking patterns of four HLA alleles (HLA-B x 5101, HLA-B x 1501, HLA-A x 02:06 and HLA-B x 57:01) against four commercial drugs.

Methodology

Four drugs (phenytoin (PHT), amoxicillin (AMX), aceclofenac (ACE) and ciprofloxacin (CIP)) were investigated for their docking behavior against four HLA alleles (HLA-B x 5101, HLA-B x 1501, HLA-A x 02:06, and HLA-B x 57:01) using the SwissDock method. In addition, toxicity (Tox) and the search tool for interactions of chemicals (STITCH) (protein-drug interaction) analyses were also carried out using the predicating the small molecule pharmaco-kinetic (pk) properties using graph-based signature method (pkCSM) and STITCH free online servers, respectively.

Results

Toxicity analysis showed that two drugs (amoxicillin and ciprofloxacin) exhibit hepatotoxicity. The STITCH analysis of the drug amoxicillin revealed its interaction with two human proteins. The drug phenytoin exhibited the lowest binding energy (LBE) with all four HLA alleles (HLA-B x 5101, HLA-B x 1501, HLA-A x 02:06, and HLA-B x 57:01).

Conclusions

The present findings provide new knowledge about the four drugs (phenytoin (PHT), amoxicillin (AMX), aceclofenac (ACE) and ciprofloxacin (CIP)) and their binding affinities with HLA alleles, which may cause cutaneous adverse drug reactions.

## Introduction

Stevens-Johnson syndrome (SJS) and toxic epidermal necrolysis (TEN) are two rare, life-threatening allergic reactions (rxns) induced mainly due to the usage of drugs that affect both the skin and mucous membranes [1]. Three variants of epidermal necrolysis (EN) are (i) SJS, which is characterized by the involvement of less than 10% of the body surface area; (ii) SJS-TEN overlaps, which are characterized by the involvement of 10-30% of the body surface area, and (iii) TEN, which is characterized by the involvement of more than 30% of the body surface area [2, 3]. The reported major risk factors for SJS and TEN include (i) possessing a slow acetylator genotype, which has been associated with accelerated susceptibility to certain autoimmune diseases (ADs), (ii) the presence of immune-suppression, which describes a weak prognosis in the advancement of SJS/TEN, (iii) usage of anti-convulsant agents concurrent with radiotherapy and (iv) possessing specific human leukocyte antigen (HLA) alleles like HLA-B x 15:02, HLA-A x 31:01 and HLA-B x 58:01 [4]. Moreover, the recent understanding of EN is that it is an immune-linked pathway mediated by granylysin, which in turn releases drug-specific cluster of differentiation 8 (CD8) cytotoxic T cells and natural killer cells (NKC) [5]. Furthermore, the death of keratinocytes is stimulated by cytotoxic CD8 T cells and NKC via interacting with the HLA and drug antigens [6]. Approximately 5-20% of SJS and TEN cases remain idiopathic. Moreover, SJS and TEN are mainly caused by a combination of immune predisposition and exogenous stimuli such as drug/medication or infection that results in the apoptosis of epithelial cells. Furthermore, drug/medication exposure is associated with 50-95% of the cases, depending on the population examined. Especially, people with certain HLA serotypes, T-cell receptor (TCR) subtypes or differences in their ability to absorb, distribute to tissues, metabolize or excrete drugs/medications have a higher probability of developing SJS and TEN. In a nutshell, the pathophysiology of SJS and TEN has been evidenced by the involvement of acute inflammatory vesiculo-bullous reaction of the (a) mucosa of the ocular surface, (b) oral cavity, (c) genitals and (d) the skin and immune-associated reactions from both innate and adaptive immune systems [7].

The most common drugs that cause SJS and TEN include abacavir, aceclofenac, albendazole, allopurinol, aminopenicillin, amlodipine, amoxycillin, barbiturates, carbamazepine, cephalosporin, chloroquine, ciprofloxacin, cotrimoxazole, dapsone, diclofenac, doxycycline, ethambutol, erythromycin, felbamate, fluconazole, fluoroquinolone, homeopathic medicines, ibuprofen, isoniazid, ketoprofen, lamotrigine, levofloxacin, naproxen, nevirapine, oxicam, paracetamol, phenytoin, piroxicam, promethazine, rifampicin, rofecoxib, sulfasalazine, sulfadiazine, sulfadoxine, sulindac, thiacetazone, valdecoxib and vancomycin [1, 2, 3, 8-11]. Based on the literature survey, four drugs were selected: (a) an anti-epileptic/anti-convulsant drug (phenytoin, PHT), (b) antibiotic drugs (amoxicillin (AMX) and ciprofloxacin (CIP)) and (c) a non-steroidal anti-inflammatory drug (aceclofenac (ACE)). These four drugs were subjected to docking analysis against four HLA alleles, namely (i) HLA-B x 5101, (ii) HLA-B x1501, (iii) HLA-A x 02:06 and (iv) HLA-B x 57:01 using the SwissDock method.

## Materials and methods

Ligand preparation

The chemical structures of the four drugs - (a) phenytoin (PubChem ID 1775), (b) amoxicillin (PubChem ID 33613), (c) aceclofenac (PubChem ID 71771) and (d) ciprofloxacin (PubChem ID 2764) - and one reference drug, abacavir (PubChem ID 441300), were obtained from PubChem compound database. All four drugs were depicted using ChemDraw 2D and 3D and further subjected to SwissDock analysis [12].

Toxicity (Tox) analysis

Toxicity (Tox) analysis of the four chosen drugs was performed using the predicating the small molecule pharmaco-kinetic (pk) properties using graph-based signature method (pkCSM) free web server [13].

STITCH (protein-drug interaction) analysis

The four chosen drugs (PHT, AMX, ACE and CIP) were assessed for their interaction with the target human proteins using the search tool for interactions of chemicals (STITCH) free web server [14].

Identification and preparation of target proteins

The 3D structures of human leukocyte antigen (HLA-B x 5101; PDB ID: 1E27 with a resolution of 2.20 Aᵒ), human leukocyte antigen (HLA-B x 1501; PDB ID: 1XR9 with a resolution of 1.79 Aᵒ); human leukocyte antigen (HLA-A x 02:06; PDB ID: 3OXR with a resolution of 1.70 Aᵒ) and human leukocyte antigen (HLA-B x 57:01; PDB ID: 3VRI with a resolution of 1.60 Aᵒ) were retrieved from Protein Data Bank (PDB) database. A chain of these proteins was prepared individually by removing other chains, ligands and even crystallographically observed water molecules by applying UCSF Chimera software tool [12].

Docking study

A docking investigation was carried out for the four selected drugs using a SwissDock free web server. Finally, the binding site interaction analysis was performed by using PyMOL software tool [13].

Docking procedure validation

The validation of the docking procedure was done using abacavir (Aba) as a reference drug. Moreover, the root mean square deviation (RMSD) analysis of all the docked complexes (four drugs) was individually compared with that of abacavir docked complex (reference drug) for each selected protein through PyMOL using the "align" command [15]. The RMSD values were utilized as a quantitative guide to know how close the prediction matches with that of the binding mode of abacavir (reference drug). The RMSD values along with visual analysis and docking scores were utilized to assess the reliability of each of the docking programs. The scores were also utilized to investigate the relationship between the binding scores and positions [15].

## Results

In the present study, toxicity analysis showed that all the four selected drugs do not exhibit any human ether-a-go-go-related gene-1 and 2 (hERG 1 and 2) inhibition activities (which are mainly involved in the repolarization of cardiac action potential in humans) (Table 1). On the other hand, two drugs (amoxicillin and ciprofloxacin) exhibit hepatotoxicity.

**Table 1:** Toxicity (Tox) analysis of the four drugs using pkCSM free web server. pkCSM, predicating the small molecule pharmaco-kinetic (pk) properties using graph-based signature method; N, No; Y, Yes; AT, AMES toxicity; hERG-1, human ether-a-go-go-related gene inhibitor-1; hERG-2, human ether-a-go-go-related gene inhibitor-2;  HT, hepatotoxicity; SS, skin sensitization; ORAT, oral rat acute toxicity (lethal dose LD50 in mol per kg;  MT, Minnow toxicity (log mM).

Ligand	AT	hERG-1	hERG-2	HT	SS	ORAT (LD_50_)	MT
Phenytoin	N	N	N	N	N	1.88	1.22
Amoxicillin	N	N	N	Y	N	1.74	4.97
Aceclofenac	N	N	N	N	N	2.38	0.83
Ciprofloxacin	N	N	N	Y	N	2.89	1.19

**Figure 1:**
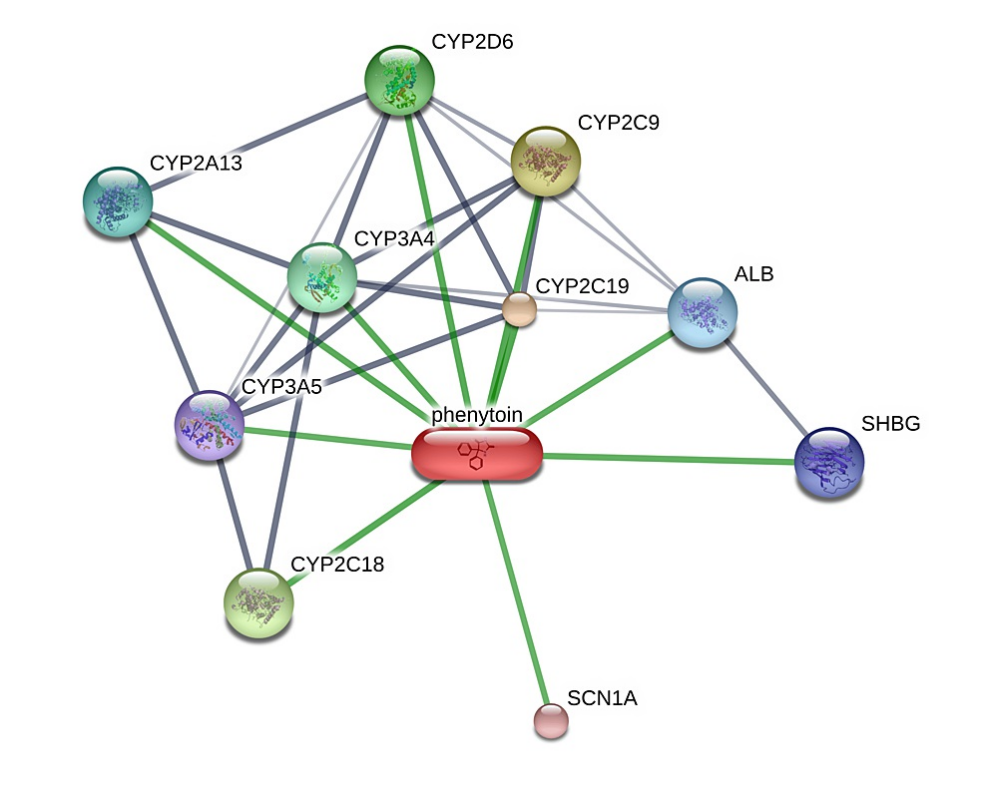
Interaction of the drug phenytoin with the human proteins depicted by STITCH free web server. Image credit: Radhakrishnan Narayanaswamy. ALB, albumin; CYP, cytochrome P450- 2A13, 2C9, 2C18, 2C19, 2D6, 3A4, 3A5; SCN1A, sodium voltage-gated channel alpha subunit 1; SHBG, sex hormone binding globulin

The STITCH analysis of the drug phenytoin shows its interaction with 10 human proteins, namely albumin (ALB), CYP (cytochrome P450 - 2A13, 2C9, 2C18, 2C19, 2D6, 3A4, 3A5), sodium voltage-gated channel alpha subunit 1 (SCN1A) and sex hormone binding globulin (SHBG) (Figure 1).

**Figure 2:**
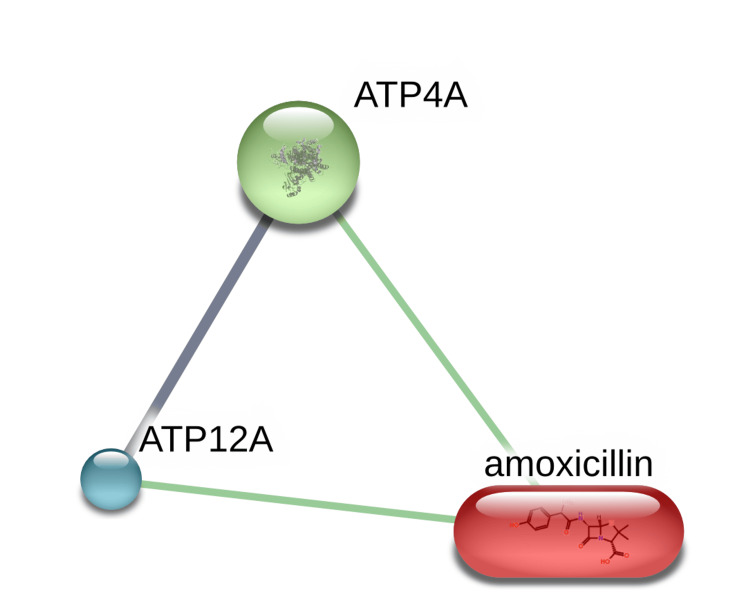
Interaction of the drug amoxicillin with the human proteins depicted using the STITCH free web server. Image credit: Radhakrishnan Narayanaswamy. ATP4A, Adenosine triphosphate synthase (ATPase) H+/K+ transporting subunit alpha; ATP12A, adenosine triphosphate synthase (ATPase) H+/K+ transporting non-gastric alpha2 subunit

The STITCH analysis of the drug amoxicillin reveals its interaction with two human proteins, namely adenosine triphosphate synthase (ATPase) H+/K+ transporting subunit alpha (ATP4A) and adenosine triphosphate synthase (ATPase) H+/K+ transporting non-gastric alpha 2 subunit (ATP12A) as shown in Figure 2.

**Figure 3:**
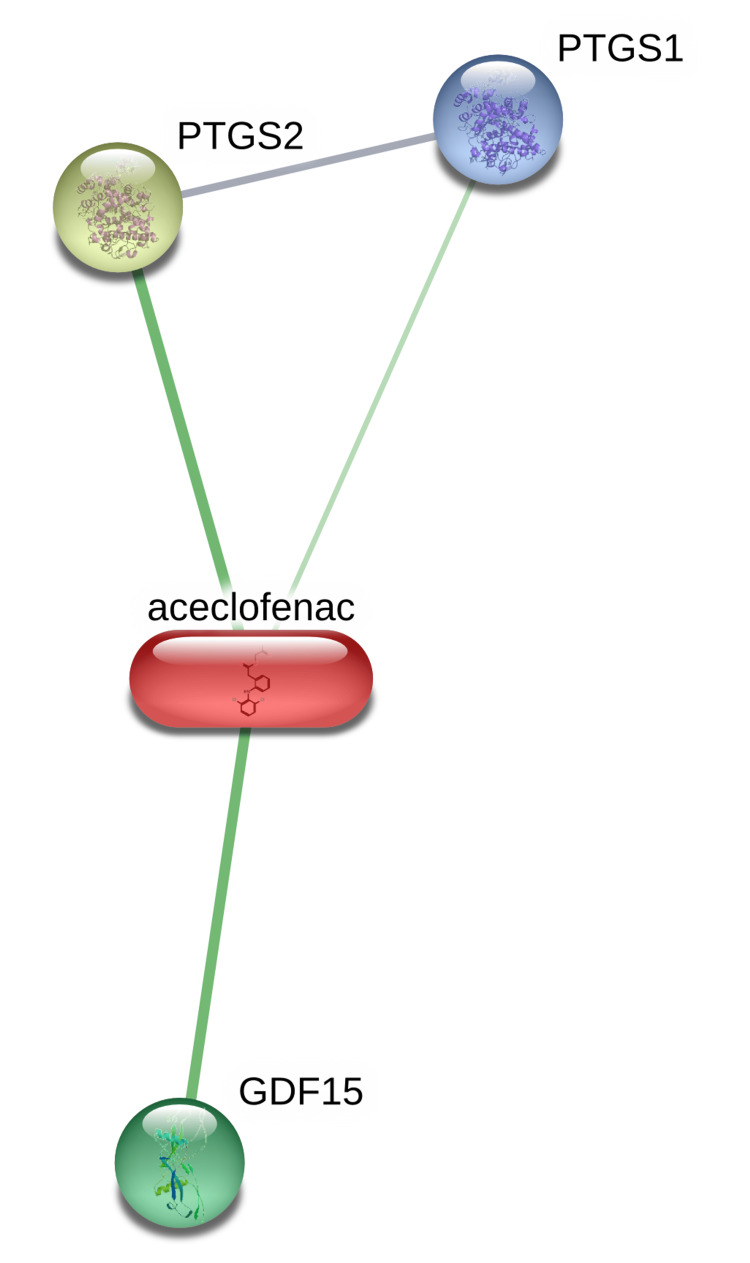
Interaction of the drug aceclofenac with the human proteins depicted using STITCH free web server. Image credit: Radhakrishnan Narayanaswamy. GDF 15, growth differentiation factor 15; PTGS1, prostaglandin G/H synthase 1 or cyclooxygenase 1; PTGS2, prostaglandin G/H synthase 2 or cyclooxygenase 2

The STITCH analysis of the drug aceclofenac demonstrates its interaction with three human proteins, namely, growth differentiation factor 15 (GDF 15), prostaglandin G/H synthase 1 or cyclooxygenase 1 (PTGS1) and prostaglandin G/H synthase 2 or cyclooxygenase 2 (PTGS2) (Figure 3).

**Figure 4:**
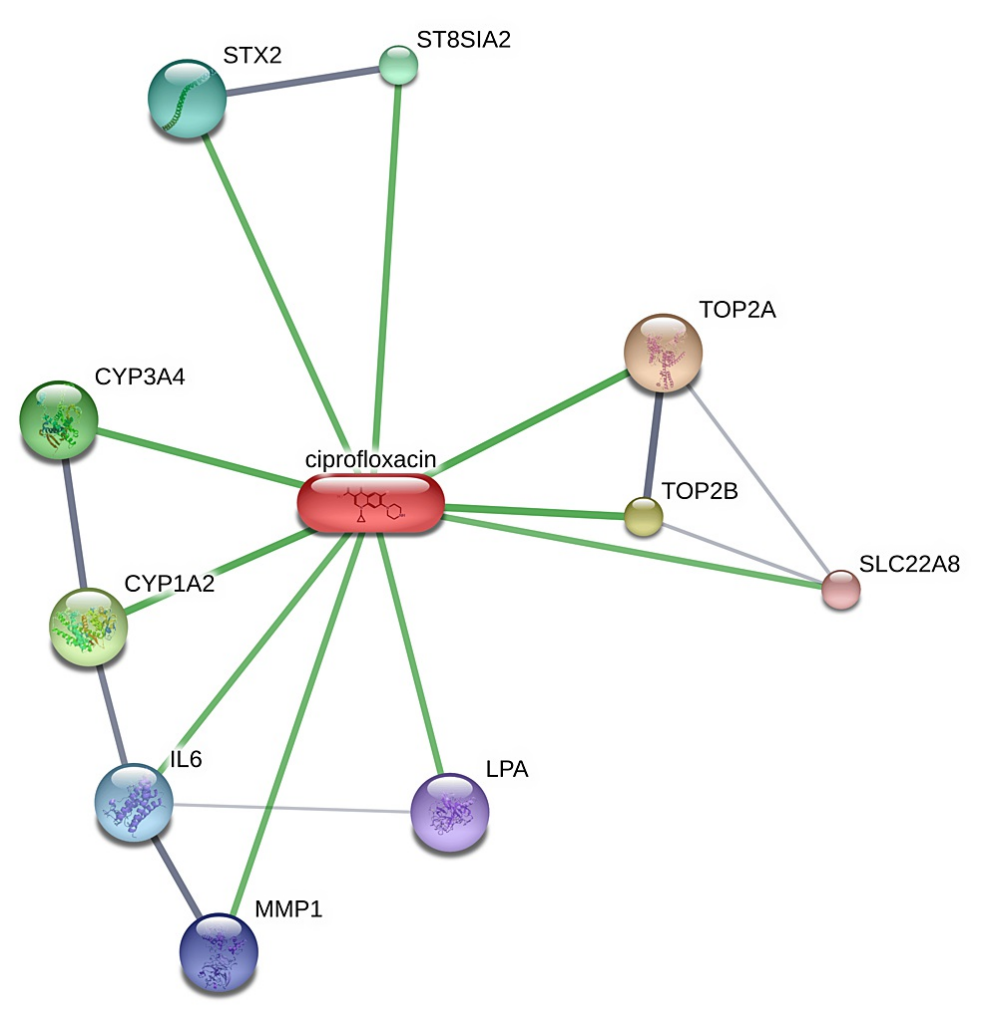
interaction of the drug ciprofloxacin with the human proteins using STITCH free web server. Image credit: Radhakrishnan Narayanaswamy. CYP, cytochrome P450-1A2, 3A4; IL-6, interleukin 6; LPA, lipoprotein (a) or low-density lipoprotein (LDL); MMP 1, matrix metalloproteinase 1; SLC22A8, solute carrier family 22 member 8; ST8SIA2, ST8 alpha-N-acetyl-neuraminide alpha-2,8-sialyl transferase 2; STX2, syntaxin 2; TOP2A, topoisomerase II alpha and TOP2B, topoisomerase II beta

The STITCH analysis of the drug ciprofloxacin confirms its interaction with 10 human proteins, namely, CYP (cytochrome P450 - 1A2, 3A4), interleukin 6 (IL-6), lipoprotein (LPA) (a) or low-density lipoprotein (LDL), matrix metalloproteinase 1 (MMP 1), solute carrier family 22 member 8 (SLC22A8), ST8 alpha-N-acetyl-neuraminide alpha-2,8-sialyl transferase 2 (ST8SIA2), syntaxin 2 (STX2), topoisomerase II alpha (TOP2A) and topoisomerase II beta (TOP2B) (Figure 4).

The docking analysis showed that ciprofloxacin demonstrates a maximum binding energy (MBE) of -8.13 kcal per mol with the human leukocyte antigen (HLA-B x 5101). In contrast, phenytoin has the least binding energy (LBE) of -7.28 kcal per mol with HLA-B x 5101 (Table 2). Phenytoin and amoxicillin interact with the Tyr7 amino acid residue (AAR) of the human leukocyte antigen (HLA-B x 5101) (Table 2).

**Table 2:** SwissDock binding energy analysis of four drugs with human leukocyte antigen (HLA-B x 5101) using the SwissDock method. Note: *RMSD, root mean square deviation; **Abacavir, reference drug.

Drug name	SwissDock binding energy (kcal/mol)	Interactions of amino acid residues	Bond distance (A^ᵒ^)	RMSD^*^ compared with abacavir docked complex (A^ᵒ^)
Phenytoin	-7.28	Tyr7; His171; Tyr59	3.3; 3.0; 2.8	0.76
Amoxicillin	-8.03	Tyr7; Tyr9; Asn70; Tyr159	2.4; 2.9; 3.2 and 3.2; 3.1	0.62
Aceclofenac	-7.37	No binding	-	0.68
Ciprofloxacin	-8.13	Asn63	3.4	0.83
Abacavir^**^	-7.87	Tyr159; Trp167	3.4; 3.2	

The root mean square deviation (RMSD) analysis reveals that if RMSD is less than or equal to 2.0 Aᵒ, it is indicated as “excellent”, whereas if RMSD greater than 2.0 Aᵒ and less than 3.0 Aᵒ, then it is considered as “fair". On the other hand, if RMSD is greater than or equal to 3.0 Aᵒ, it is indicated as “poor”.

The docking investigation showed that amoxicillin demonstrated an HBE of -8.41 kcal per mol with human leukocyte antigen (HLA-B x 1501). In contrast, phenytoin (PHT) exhibited the lowest binding energy (LBE) (-6.99 kcal per mol) with HLA-B x 1501 (Table 3). Three drugs (phenytoin, amoxicillin and aceclofenac) interact with the Tyr9 amino acid residue (AAR) of the human leukocyte antigen (HLA-B x 1501) (Table 3).

**Table 3:** SwissDock binding energy analysis of the four drugs with human leukocyte antigen (HLA-B x 1501) using the SwissDock method. Note: *RMSD, root mean square deviation; **Abacavir, reference drug.

Drug name	SwissDock binding energy (kcal/mol)	Interactions of amino acid residues	Bond distance (A^ᵒ^)	RMSD^*^ compared with abacavir docked complex (A^ᵒ^)
Phenytoin	-6.99	Tyr9; Asn70; Tyr74	2.2; 3.5; 2.8	0.88
Amoxicillin	-8.41	Tyr9; Asn70; Tyr74; Tyr99; Glu152	3.0; 3.1; 2.9; 3.4 and 3.4; 2.1	1.24
Aceclofenac	-8.19	Tyr9; Arg62; Glu63; Tyr99	3.2; 3.3; 1.8; 3.3 and 3.1	0.70
Ciprofloxacin	-7.74	Tyr74; Arg97; Gln155	3.0; 3.5; 3.2	1.76
Abacavir^**^	-8.17	Tyr7; Tyr9; Glu63; Tyr99	2.5; 3.4; 3.3; 3.2	

The docking analysis showed that ciprofloxacin demonstrates an MBE of -7.83 kcal per mol with human leukocyte antigen (HLA-A x 02:06). In contrast, phenytoin displays an LBE of -7.13 kcal per mol with HLA-A x 02:06 (Table 4). Two drugs (aceclofenac and ciprofloxacin) interact with Arg97 and His114 amino acid residues (AAR) of the human leukocyte antigen (HLA-A x 02:06) (Table 4).

**Table 4:** SwissDock binding energy analysis of the four drugs with human leukocyte antigen (HLA-A x 02:06) using the SwissDock method. Note: *RMSD, root mean square deviation; **Abacavir, reference drug.

Drug name	SwissDock binding energy (kcal/mol)	Interactions of amino acids residues	Bond distance (A^ᵒ^)	RMSD^*^ compared with Abacavir docked complex (A^ᵒ^)
Phenytoin	-7.13	Trp51; Thr178	3.4; 3.3	2.66
Amoxicillin	-7.64	Thr31; Arg48; Thr178; Arg181	2.3; 3.0, 3.2 and 3.4; 2.6; 2.6	3.04
Aceclofenac	-7.56	Arg97; His114	3.2, 3.4 and 3.4; 2.1	1.01
Ciprofloxacin	-7.83	Arg97; His114	3.3; 3.5	0.93
Abacavir^**^	-7.84	Tyr99	3.3	

The docking investigation showed that ciprofloxacin demonstrated an HBE of -8.40 kcal per mol with human leukocyte antigen (HLA-B x 57:01). In contrast, phenytoin exhibited an LBE of -6.67 kcal per mol with HLA-B x 57:01 (Table 5). Ciprofloxacin interacts with Tyr123 amino acid residue (AAR) of the human leukocyte antigen (HLA-B x 57:01) (Table 5).

**Table 5:** SwissDock binding energy analysis of the four drugs with human leukocyte antigen (HLA-B x 57:01) using the SwissDock method. Note: *RMSD, root mean square deviation; **Abacavir, reference drug.

Drug name	SwissDock binding energy (kcal/mol)	Interactions of amino acids residues	Bond distance (A^ᵒ^)	RMSD^*^ compared with Abacavir docked complex (A^ᵒ^)
Phenytoin	-6.67	Gly237; Arg239; Thr240	3.0; 3.1; 3.0	2.88
Amoxicillin	-7.99	Glu63; Asn66; Tyr159; Tyr171	1.9; 3.1; 2.8; 2.2	1.82
Aceclofenac	-7.69	Tyr74; Asn77	3.3; 3.2	0.71
Ciprofloxacin	-8.40	Tyr123	3.2	0.96
Abacavir^**^	-8.00	No binding	-	

## Discussion

In the present study, the toxicity (Tox) analysis of the two drugs amoxicillin (AMX) and ciprofloxacin (CIP) showed that they exhibit hepatotoxicity. The current finding correlates with the previous report, in which both the drugs AMX and CIP displayed hepatotoxicity in an in vivo (rat) experiment [16]. The association of cytochrome P450 2C9 x 3 (CYP2C9 x 3) and human leukocyte antigen (HLA-B x 51:01) allele in phenytoin (PHT)-induced cutaneous adverse drug reactions was reported in South Indian Tamil patients [17]. The association between the human leukocyte antigen (HLA-B x 15:02) allele and carbamazepine-induced SJS was observed in Han Chinese patients [5]. Yang and co-workers (2009) noted that abacavir showed good binding affinities towards HLB-B x 57:01 using the molecular docking approach [18]. The association between the human leukocyte antigen (HLA-B x 1502) allele and carbamazepine-induced SJS was noted in Indian patients [19]. Similarly, the association between the human leukocyte antigen (HLA-B x 5801) allele and allopurinol-induced SJS and TEN was reviewed by Somkrua and colleagues in 2011 [20]. Moreover, in silico risk assessment of human leukocyte antigen (HLA-A x 02:06) associated SJS and TEN caused by various ingredients present in cold medicine in Japan was reported by Isogai and colleagues in 2013 [21]. The strong association of human leukocyte antigen (HLA-B x 15:21) allele and carbamazepine-induced SJS was reported in Asian patients [22]. The association between the human leukocyte antigen (HLA-B x 15:02) allele and carbamazepine-induced SJS and TEN was shown in Malaysian patients [23]. Ramsbottom and colleagues (2018) used two different HLA risk alleles (HLA-B x 15:02 and HLA-B x 57:01) for docking studies [15]. A strong association between the human leukocyte antigen (HLA-B x 13:01) allele and dapsone-induced SJS and TEN was observed in Thai and Taiwanese patients [24]. Jiang and co-workers (2023) noted that levofloxacin showed good binding affinities towards HLA-B x 13:01 and HLA-B x 13:02 compared to those of HLA-B x 46:01 using the molecular docking approach [11].

Limitations and future recommendations

The findings of the present investigation are based on molecular docking analysis, which provides new insight into four drugs (phenytoin (PHT), amoxicillin (AMX), aceclofenac (ACE) and ciprofloxacin (CIP)) and their interactions with four different human leukocyte antigen alleles (HLA-B x 5101, HLA-B x 1501, HLA-A x 02:06 and HLA-B x 57:01). However, biochemical, cell-based assays and animal experiments are needed to confirm the association of HLA alleles with adverse drug reactions (ADR).

## Conclusions

In the current investigation, all four drugs (phenytoin, amoxicillin, aceclofenac and ciprofloxacin) have shown the potential to dock with all four targeted human leukocyte antigen alleles (HLA-B x 5101; HLA-B x 1501; HLA-A x 02:06 and HLA-B x 57:01). Moreover, two drugs (amoxicillin and ciprofloxacin) exhibit hepatotoxicity. Hence, the current investigation has highlighted a new understanding of the four drugs and their binding affinities with human leukocyte antigen (HLA) alleles, which may cause cutaneous adverse drug reactions.
